# Supplementary material for: Crowding in or crowding out? How local government debt influences corporate innovation for China
Source: PLoS One. 2021 Nov 8;16(11):e0259452. doi: 10.1371/journal.pone.0259452 (PMC8575287; doi:10.1371/journal.pone.0259452)
Supplement: Attachment — Submitted filename: Response to Reviewers.docx [file pone.0259452.s002.docx]

**Responses to Reviewer’s Comments**

*This study examines the effects of local government debt on corporate R&D activities by focusing on the case of China. The authors find the crowding-out effects, and they argue that three channels (consumption of fiscal resources, strengthening tax collection efforts, consumption of credit resources) are channels that lead to these results. While the study tackles the interesting question with a valid empirical framework, I believe that there is much room for the improvement of the current document. Here, I mainly provide four suggestions.*

*1.* ***Institutional Background***

*For international readers with various backgrounds, I recommend the authors elaborate on the institutional background of the study. For example, how and why does the corporate's innovative strategy require the local government's fiscal policies in China? For example, private enterprises’ R&D activities are not necessarily dependent on government funding in many developed countries.*

*To do so, I suggest that the authors add a new section—Institutional Background—before (or after) the literature review to explain more about the detailed system in China. Providing anecdotes or examples can be beneficial. While some of the relevant information is included in the theoretical hypothesis in the current version, it would be effective to separate the hypothesis developments with institutional background.*

**⇒Response:**

We thank the reviewer for carefully reviewing our manuscript. The lack of an introduction to the institutional background of the study has indeed brought some doubts to international readers of various backgrounds. We totally agree with your opinion. **Therefore, we have added an explanation of the background of the Chinese system (2. Institutional Background) before the Literature Review section.**

The specific content added in the revised manuscript is as follows.

2. Institutional Background

Since 1978, with the reform and opening policy, China has achieved rapid economic growth by relying on cheap labor and investment in infrastructure construction [1]^[1]^. However, with the recent rise in labor costs, this growth model is considered outdated [2]^[2]^. The country’s top leaders are promoting innovation as the key to sustained economic growth [3]^[3]^. Further, the country’s R&D investment has risen from 10,240 billion yuan in 2012 to 24,426 billion yuan in 2020. As shown in Figure 1, the proportion of R&D investment in GDP has been increasing yearly.

**Fig 1. Chinese government innovation investment.**

Billion yuan

| Year | R&D investment |
| --- | --- |
| 2012 | 10240 |
| 2013 | 11906 |
| 2014 | 13312 |
| 2015 | 14220 |
| 2016 | 15500 |
| 2017 | 17500 |
| 2018 | 19657 |
| 2019 | 21737 |
| 2020 | 24426 |

|  | the proportion of R&D investment in GDP |
| --- | --- |
| 2012 | 1.97% |
| 2013 | 2.09% |
| 2014 | 2.09% |
| 2015 | 2.1% |
| 2016 | 2.08% |
| 2017 | 2.12% |
| 2018 | 2.18% |
| 2019 | 2.19% |
| 2020 | 2.4% |

In China’s “13th Five-Year Plan (The Five-Year Plan for national economic and social development is one of the most important plans of the government. It records the national strategy during this period),” innovation is regarded as the primary guiding principle of the economic policy. Since strong economic intervention and command characteristics exist in the country, local governments have actively adopted various fiscal policies, such as fiscal subsidies, transfer payments, loan policy, and tax incentives, to support innovative activities. Specifically, unlike some developed countries, owing to China’s imperfect financial system and intellectual patent protection laws, the government’s fiscal policy for corporate innovation activities becomes more important.

China’s financial system is underdeveloped [4]^[4]^. It comprises the banking and intermediation sector, financial markets, shadow financial sector, and foreign sectors [5]^[5]^. Compared with other developed countries and emerging economies, China’s financial system has always been dominated by a large banking system [5]. Bank loans remain the most important financing channel for innovation in the country. However, banks are more inclined to provide loans to large enterprises, especially state-owned enterprises (SOE) [6]^[6]^, as they believe that this type of enterprise has less risk of repayment and can obtain strong government assistance [7]^[7]^. Conversely, most Small and medium enterprises (SMEs) and non-state-owned enterprises (NSOE) are difficult to obtain funds for innovation activities from banks. Although China’s stock and bond markets are established, the conditions for companies to go public and requirements for issuing bonds remain quite strict. Thus, most corporates cannot obtain funds from the financial market. The shadow financial and foreign sectors are not sufficiently large to provide enterprises with adequate and continuous funding for innovative activities, and their costs are generally relatively high. Therefore, the government’s fiscal policy is essential to address the financing constraints of corporate innovation activities.

China’s laws on the protection of intellectual property rights are weaker than that of some developed countries [3]. Thus, the corporates’ innovation benefits are difficult to guarantee. Owing to the high cost and uncertainty of the benefits of innovation, corporates have no incentive to carry out such activities. The Chinese government cannot ameliorate its financial system and patent protection laws in the short term. Thus, it must adopt some fiscal policies to encourage enterprises to carry out innovative activities to maintain sustained economic growth.

***2. Causality argument***

*Considering that the local government’s debts are non-random, inferring a causal linkage is difficult in this study. Of course, the authors conduct many robustness checks to support their results, which should be applauded for their efforts. Still, I have some reservations and request the following tests to improve the quality of estimation.*

*Dynamic panel analysis suggested by Arellano and Bond (1991) would be advantageous as the dataset is longitudinal and rich. This test is also helpful considering that R&D in the past may affect the current decision-making because R&D activities are performed on the basis of a long-term perspective.*

**⇒Response:**

Thank you for raising this question. As you have mentioned, the dependent variable corporate R&D investment may indeed have a lag effect. That is, the corporate’s past R&D investment may have an impact on the current decision-making, which may lead to biases in the estimated results of this study. **To address this problem, we have followed your recommendations and added a dynamic panel data model to the robustness test of the revised manuscript.**

The specific content added in the revised manuscript is as follows.

In the revised manuscript, we have added a one-period lagging variable of the dependent variable corporate R&D investment (${RD}_{p,i,t-1}$) on the basis of the original benchmark model, as expressed in formula (9). The current empirical model is a dynamic panel model, and the use of fixed-effect estimation methods will lead to inconsistencies in parameter estimates. Therefore, the system GMM is employed to estimate formula (9) to ensure the accuracy of the results.

${RD}_{p,i,t}=\alpha_{4}+\beta_{4}{LGD}_{p,t-1}+\sigma_{4}{RD}_{p,i,t-1}+\gamma_{4}{Control}_{p,i,t}+\delta_{t}+\theta_{p}+\tau_{ind}+\varepsilon_{p,i,t}$ (9)

The estimation results are presented in column (1) of Table 9. We observe that the coefficient of the one-period lagging variable of corporate R&D investment (${RD}_{p,i,t-1}$) is significantly positive. and that of the local government debt ($\mathrm{LGD}$) is significantly negative. This result implies that previous corporate R&D investment has a positive impact on current R&D activities. However, it does not affect the main conclusion of this study: LGD reduces corporate R&D investment. Further, to ensure the robustness of the results, we have successively added the two-period and three-period lagging variables of corporate R&D investment in formula (9). The results are shown in columns (2) and (3) in Table 9. It can be observed that the influence of corporate past R&D investment on the current decision-making gradually weakens. The three-period lagging variable of corporate R&D investment in column (3) have no significant impact on the current R&D decision-making. Further, in columns (2) and (3) of Table 9, the coefficients of LGD have remained significantly negative, thus indicating that the LGD has reduced the corporate R&D investment. This finding is consistent with these conclusions and ensures the robustness of the benchmark estimation results.

The validity of the system GMM estimation results must meet two conditions: the random error term does not have serial correlation, and the weak instrumental variable problem does not exist. The corresponding AR (1), AR (2), and Hansen statistics are shown in columns (1), (2), and (3) of Table 9. Clearly, AR (1) is significant at the 1% level, and AR (2) is not significant, thereby implying that no serial correlation exists in the random error term in the model. The Hansen statistics show that the null hypothesis cannot be rejected, thus implying that the weak instrumental variable problem does not exist in the models. Therefore, these results are valid.

| **Table 9. The estimation results of the system GMM.** | | | |
| --- | --- | --- | --- |
| **Variables** | **Dependent variable: RD** | | |
|  | **SYS-GMM** | **SYS-GMM** | **SYS-GMM** |
|  | **(1)** | **(2)** | **(3)** |
| LGD | -0.003** | -0.002** | -0.002** |
|  | (-2.405) | (-2.367) | (-2.367) |
| L.RD | 0.510*** | 0.462*** | 0.439** |
|  | (7.831) | (4.602) | (2.424) |
| L2.RD |  | 0.158* | 0.123* |
|  |  | (1.824) | (1.845) |
| L3.RD |  |  | 0.039 |
|  |  |  | (0.615) |
| Other controls | Yes | Yes | Yes |
| Provincial fixed effects | Yes | Yes | Yes |
| Industry fixed effects | Yes | Yes | Yes |
| Year fixed effects | Yes | Yes | Yes |
| Observations | 10950 | 8024 | 5742 |
| AR(1) | -7.613*** | -5.742*** | -5.041*** |
|  | 0.000 | 0.000 | 0.000 |
| AR(2) | -0.441 | 1.173 | 3.180 |
|  | 0.660 | 0.242 | 0.520 |
| Hansen test | 5.81 | 7.93 | 11.82 |
|  | 0.214 | 0.474 | 0.365 |
| **Notes:** ***, **, and * are indicate significant levels of 1%, 5%, and 10% levels, respectively. T-statistics are provided in parentheses below each coefficient estimate. Other controls include LnGDP, LnPop, SIZE, SOE, AGE, LEV, ROA, FIX, INV, and SHR1. | | | |

In fact, the estimation results of the dynamic panel model are consistent with the original manuscript. However, we did not take the dynamic panel model as a benchmark model mainly owing to two concerns. First, the research data in this paper are the short-panel data structure (N=2926, T=7), which is different from the long panel because the time span of the short-panel data is shorter and the dynamic effect is generally weak. Second, endogenous issues are considered. In the dynamic panel model, the lagged dependent variable is also endogenous. Thus, considering the endogenous issue of both the core explanatory variable and the lagged dependent variable simultaneously is necessary. This step will increase the complexity of the empirical analysis and reduce the robustness of the estimation results. Therefore, we mainly focused on static panel models in empirical analysis and placed dynamic panel models in robustness tests.

*While the authors apply the IV strategy to resolve the endogeneity, I am not convinced whether the current IV is satisfied with the exclusion assumption. There is no explanation about how the IV can resolve the endogeneity—such as reverse causality. For example, we can imagine that R&D activities may improve provincial economic conditions (such as employment, etc.) which may lead to affecting the debts of local governments, which the authors do not provide any explanation of how the IV can address this issue.*

*If the IV is not sufficiently convinced, I suggest that the authors should remove any causal argument in the current document. Instead, focusing on the correlation between the local governments’ debt and R&D activities can be better, which still contributes to the literature. (I admit that it requires substantial revisions, but it is worthwhile.)*

**⇒Response:**

Thank you very much for your comments. We apologize that we did not elaborate and demonstrate the endogenous problems of the empirical model and the rationality of the instrumental variables in the original manuscript. **To address the problems raised by the reviewers, in the revised manuscript, we present in detail how this paper alleviated the endogenous problem and the rationality of the instrumental variables.**

The causes of endogenous problems mainly include the following: measurement errors, omitted variables, and reverse causality [8]^[8]^. Regarding measurement errors, as proxy variables reflecting the intensity of enterprise R&D investment and the repayment of LGD, $\mathrm{RD}$ and $\mathrm{LGD}$have certain noises, and measurement errors may exist. For example, a firm may falsely report on R&D investment to obtain fiscal subsidies, or the debt level of a local government measured in this study ignores some other debts. Therefore, due to measurement errors of core explanatory and dependent variables, the estimation results of this study may be biased. Regarding the omitted variables, although this study controls for many regional and firm-related variables, it may still omit the impact of other regional innovation subsidy policies and industry cycles on corporate R&D investment, leading to the endogeneity of the model. Regarding reverse causality, as mentioned by the reviewer, corporate R&D investment may affect the economic development of a region, which, in turn, affects the LGD. Therefore, this study has the possibility of endogenous problems owing to reverse causality. We will reply with specific handling methods based on the three sources of the endogenous problem.

First, for the endogenous problem caused by measurement errors, we adopted other measurement methods of core variables (RD and LGD) to alleviate endogeneity. For the dependent variable corporate R&D investment indicators, we used three alternative measurement methods of $RD2$, $RD3$, and $RD4$ in terms of robustness. The empirical results presented in columns (1), (2), and (3) of Table 8 still indicate that the LGD has reduced corporate R&D investment. In addition, we take $LGD2$ as an alternative measure of the core explanatory variable LGD. Further, the results shown in column (4) of Table 8 support these conclusions. For the convenience of the reviewers, we have reported these results directly. Therefore, to a certain extent, the measurement of the core explanatory variable and the explained variable can alleviate the endogenous problem caused by the measurement error; that is, the endogenous problem owing to measurement error in this study is relatively weak.

| **Table 8. Robust Test 1.** | | | | | | |
| --- | --- | --- | --- | --- | --- | --- |
| **Variables** | **RD2** | **RD3** | **RD4** | **RD** | | |
|  | **(1)** | **(2)** | **(3)** | **(4)** | **(5)** | **(6)** |
| LGD | -0.145*** | -0.002* | -0.016** |  | -0.002*** | -0.001*** |
|  | (-2.623) | (-1.875) | (-2.545) |  | (-3.921) | (-2.691) |
| LGD2 |  |  |  | -0.001** |  |  |
|  |  |  |  | (-2.486) |  |  |
| Other controls | Yes | Yes | Yes | Yes | Yes | Yes |
| Province fixed effects | Yes | Yes | Yes | Yes | Yes | No |
| Industry fixed effects | Yes | Yes | Yes | Yes | Yes | No |
| Year fixed effects | Yes | Yes | Yes | Yes | Yes | Yes |
| Industry*year fixed effects | No | No | No | No | Yes | No |
| Firm fixed effect | No | No | No | No | No | Yes |
| Observations | 14314 | 14314 | 9251 | 14314 | 14305 | 14099 |
| Adj_R^2^ | 0.564 | 0.383 | 0.385 | 0.318 | 0.316 | 0.848 |
| **Notes:** ***, **, and * are indicate significant levels of 1%, 5%, and 10% levels, respectively. T-statistics are provided in parentheses below each coefficient estimate. Other controls include LnGDP, LnPop, SIZE, SOE, AGE, LEV, ROA, FIX, INV, and SHR1. | | | | | | |

Second, in the benchmark model, this study adds many control variables at the regional and enterprise levels. It controls regional, industry, and year fixed effects to alleviate the endogenous problems caused by omitted variables. We have also made some efforts to address the endogenous problem caused by omitted variables in the robustness test. First, referring to Matsa [9]^[9]^, this study controls for the fixed effects of the industry and year interaction term to eliminate the interference of unobserved time-varying heterogeneity across industries, such as demand shocks in different years or industry life cycle changes. The empirical results are presented in column (5) of Table 8. Second, to eliminate the intervention of the omitted variables that do not change with enterprises or time, such as innovation culture of the enterprise, this study controls for the firm and year fixed effects. The empirical results are shown in column (6) of Table 8. Finally, we have added other control variables that may affect the corporate R&D investment, such as the ratio of the transferred land revenue to fiscal expenditure (TLR), that of the issuance amount of local government bonds to fiscal expenditure (LGB), and that of the issuance amount of Municipal Bonds to fiscal expenditure (CB). The empirical results are presented in column (3) of Table 10. These estimation results indicate that the core explanatory and explained variables have a significant negative correlation. For the convenience of the reviewers, we have reported these results directly as follows. Therefore, to a certain extent, the endogenous problem caused by the omitted variable can be alleviated by adding the control variables and the related fixed effect; that is, the endogenous problem of the omitted variable in this study is relatively weak.

| **Table 10. Robust Test 2.** |  |
| --- | --- |
| **Variables** | **RD** |
|  | **(3)** |
| LGD | -0.002*** |
|  | (-4.072) |
| TLR | -0.009* |
|  | (-1.837) |
| LGB | 0.001 |
|  | (0.198) |
| CB | -0.004 |
|  | (-0.353) |
| Other controls | Yes |
| Provincial fixed effects | Yes |
| Industry fixed effects | Yes |
| Year fixed effects | Yes |
| Observations | 14303 |
| Adj_R^2^ | 0.318 |
| **Notes:** ***, **, and * are indicate significant levels of 1%, 5%, and 10% levels, respectively. T-statistics are provided in parentheses below each coefficient estimate. Other controls include LnGDP, LnPop, SIZE, SOE, AGE, LEV, ROA, FIX, INV, and SHR1. | |

Finally, regarding the endogenous problem caused by reverse causality mentioned by the reviewer, which may enable this study to draw relevant conclusions. In this regard, we have discussed in detail the reverse causality in this paper from the following two aspects: one is the result of using instrumental variables and the degree of bias of the estimated results caused by reverse causality, and the other is to explain that instrumental variables are convincing.

LGD is a variable at the macro-regional level, while the enterprise R&D investment is a variable at the micro-enterprise level. Generally, the decisions of local micro-enterprises are more susceptible to macroeconomic activities, while a local micro-enterprise hardly affects the macro-level economic behavior. Therefore, from this perspective, serious reverse causality does not exist. Even if a local corporate’s R&D investment can affect the macro government debt repayment. A decrease in corporate R&D investment has a negative impact on the level of regional economic development, consequently leading to an increase in LGD and ultimately strengthening the negative impact on corporate R&D investment. Therefore, without considering reverse causality, the conclusion of the benchmark regression estimation will underestimate the actual impact of LGD on corporate R&D investment, which does not affect the main conclusion of this study.

On the other hand, to further alleviate potential endogeneity, we select the sum of the maturity of all $t-1$ Municipal Bonds as the instrument variable. From a correlation perspective, LGD in year $t-1$ clearly contains the face value of the maturing Municipal Bonds in maturity year $t-1$ (e.g., an LGFV A issued a five-year 100 billion yuan bond in 2008 and issued a three-year 150 billion yuan bond in 2010. In 2012, the LGFV’s short-term liability contained 250 billion yuan (100+150) and bank debt). Further, we excluded bonds with a maturity of less than one year to avoid the related bond interference of debt redeemed in the current year. From an exogeneity perspective, the maturity amount of the $t-1$ Municipal Bonds is determined by the time of bond issuance; hence, the exogeneity of the maturity amount of the $t-1$ Municipal Bonds is also satisfied.

The two-step estimation results are presented in Table 4. In column (1), the coefficient of IV is significantly positive in the first IV regression, thereby suggesting that the maturity period of the amount of Municipal Bonds will be reflected in the LGFVs’ short-term liabilities. In column (3), the coefficient of LGD is significantly negative in the second regression, which indicates that LGD still crowd out corporate R&D investment after considering the endogenous problems in our model. Meanwhile, the *F* value tested by Cragg-Donald Wald is 2098.92, which is much higher than the corresponding critical value of stock-Yogo [10]^[10]^. Therefore, the instrumental variable used in this study is valid.

| **Table 4. Endogenous Test: IV regression.** | | | | | | |
| --- | --- | --- | --- | --- | --- | --- |
| **Variables** | **LGD** | | **RD** | | | |
|  | **IV First Stage** | | **IV Second Stage** | |  | |
|  | **(1)** | **(2)** | **(3)** | **(4)** | **(5)** | **(6)** |
| IV | 0.0002*** | 0.0002*** |  |  | -0.000** | -0.000 |
|  | (2.831) | (2.652) |  |  | (-2.102) | (-0.781) |
| IV2 |  | -0.041** |  |  |  |  |
|  |  | (-2.058) |  |  |  |  |
| LGD |  |  | -0.006*** | -0.006*** |  | -0.002*** |
|  |  |  | (-4.175) | (-4.203) |  | (-3.524) |
| Other controls | Yes | Yes | Yes | Yes | Yes | Yes |
| Province fixed effects | Yes | Yes | Yes | Yes | Yes | Yes |
| Industry fixed effects | Yes | Yes | Yes | Yes | Yes | Yes |
| Year fixed effects | Yes | Yes | Yes | Yes | Yes | Yes |
| Observations | 13914 | 13914 | 13849 | 13849 | 13848 | 13848 |
| Adj_R^2^ | 0.952 | 0.952 | 0.316 | 0.316 | 0.317 | 0.317 |
| Cragg-Donald Wald F statistic | 2098.92 |  |  |  |  |  |
| Sargan Test |  |  |  | 0.804 |  |  |
|  |  |  |  | (0.370) |  |  |
| **Notes:** ***, **, and * are indicate significant levels of 1%, 5%, and 10% levels, respectively. T-statistics are provided in parentheses below each coefficient estimate. Other controls include LnGDP, LnPop, SIZE, SOE, AGE, LEV, ROA, FIX, INV, and SHR1. | | | | | | |

**In the revised manuscript, the specific content explaining the credibility of IV is as follows.**

However, whether the instrumental variable can alleviate the endogeneity of the model depends on the exogeneity of the instrumental variables. The following three methods are used to demonstrate the credibility of the instrumental variable in this study.

First, we use the LGD and IV as explanatory variables simultaneously and perform regressions on corporate R&D investment. If the instrumental variable indirectly affects corporate R&D investment only through the LGD, then, in the above regression, the coefficient of the instrumental variable should not be significant when the LGD is controlled. As presented in column (6) in Table 4, the coefficient of IV is not significant, while the LGD remains significant, which is in line with our expectations. Additionally, when the corporate’s R&D investment regresses to the two separately, the coefficients of both are significant. This result implies that the instrumental variable does not directly affect corporate R&D expenditures, but only through LGD.

Second, the identification method provided by Larcker and Rusticus [11]^[11]^ is used to determine whether the instrumental variable satisfies the exogenous hypothesis. Larcker and Rusticus provide a method (page 191) to identify whether instrumental variables can still help alleviate the endogeneity problem when it does not sufficiently satisfy the exogeneity assumption (semi-endogenous) [11]. Certainly, it is essentially a qualitative identification method. The identification logic is briefly described as follows: Equations (2) and (3) are the estimators of the beta value under the OLS and IV methods, respectively. Equation (4) is calculated from equation (2) < equation (3), and equation (4) can be converted to express the regression output, that is, equation (5).

$plim {beta}_{OLS}=\beta+\frac{cov(x,u)}{var(x)}=\beta+\frac{\sigma_{u}}{\sigma_{x}}corr(x,u)$	(2)

$plim {beta}_{IV}=\beta+\frac{cov(x,u)}{cov(x,z)}=\beta+\frac{\sigma_{u}}{\sigma_{x}}\frac{corr(z,u)}{corr(x,z)}$	(3)

$\left| corr\left( z,u \right) \right|<\left| corr\left( x,z \right) \right||corr(x,u)|$	(4)

$R_{zu}^{2}<R_{xz}^{2}R_{xu}^{2}$	(5)

where $x$ is the core explanatory variable, $u$ is the residual term, $z$ is the instrumental variable, and $R_{zu}^{2}$is the R-squared of the IV one-stage regression. In equation (4), even if the correlation coefficient between the instrumental variable $z$ and the residual term $u$ is not 0 (i.e., the exogeneity assumption is not fully satisfied), provided that the correlation between the instrumental variable $z$ and the independent variable $x$ is sufficiently strong, we may obtain a better IV estimate result than OLS. It should be noted that we can not obtain the true residuals, hence, $corr\left( z,u \right)$ and $corr(x,u)$ cannot be accurately calculated. As shown in column (1) of Table 4, the R-squared for the first stage of IV is 0.952. Moreover, we directly use the estimated residual (rather than the true residual) to calculate the $corr\left( z,u \right)$ = 0.048. The gap between them is so large that the slight correlation between $x$ and $u$ can meet the requirements of equation (5). Therefore, we believe that it can obtain better results than OLS, although this instrumental variable may not fully meet the exogenous requirement.

Third, referring to Demirci et al. [12]^[12]^, on the basis of the original IV, we added provincial defense expenditure (IV2) as another instrumental variable of LGD. In terms of relevance, under the premise of limited fiscal expenditure, the higher the defense expenditure, the smaller the local government’s expenditure for debt. Further, provincial defense expenditures are relatively exogenous to local economic activities. These two instrumental variables are used to further alleviate the endogeneity caused by reverse causality. The empirical results are presented in columns (2) and (4) of Table 4. It can be observed that the value of Sargan Test cannot reject the null hypothesis; that is, both instrumental variables are exogenous.

***3. Mechanism analysis***

*I believe that the mechanism analysis is not methodologically persuasive and only provides suggestive evidence. While the authors find correlations between the local government debts and fiscal variables by adopting these fiscal factors as a dependent variable, they do not give any concrete evidence that deteriorated fiscal conditions negatively affect R&D activities.*

*Here, two alternative approaches come to my mind. First, the authors may tone down their argument by removing any concrete conclusion. (In this way, the author can address the relationship between local government debts and R&D activities, and three channels can remain as possible discussions). Second, the author can conduct the regression by interacting the terms (between local government debts and fiscal variables) and R&D activities as a dependent variable. Although this is also not sufficient, it can provide a necessary condition for the authors’ argument.*

**⇒Response:**

Thank you for raising this problem and comments. **We have followed your opinion to prove the mechanism of this study through the interaction of LGD and three intermediary variables.**

The specific content in the revised manuscript is as follows.

In the theoretical hypothesis, we analyze the three channels through which LGD affects corporate R&D investment, by consuming regional fiscal resources, strengthening government tax collection efforts, and consuming regional credit resources. Now, we further test whether this mechanism exists by introducing three interaction terms in the benchmark regression: the interaction item between LGD and fiscal subsidies, that between LGD and corporate actual tax burden, and that between LGD and external financing dependence. The models are as follows:

${RD}_{p,i,t}=\alpha_{1}+\beta_{1}{LGD}_{p,t-1}+\lambda_{1}{Sub}_{p,i,t}+\chi_{1}{LGD}_{p,t-1}*{Sub}_{p,i,t}+\gamma_{1}{Control}_{p,i,t}+\delta_{t}+\theta_{p}+\tau_{ind}+\varepsilon_{p,i,t}$	(6)

${RD}_{p,i,t}=\alpha_{2}+\beta_{2}{LGD}_{p,t-1}+\lambda_{2}{ERT}_{p,i,t}+\chi_{2}{LGD}_{p,t-1}*{ERT}_{p,i,t}+\gamma_{2}{Control}_{p,i,t}+\delta_{t}+\theta_{p}+\tau_{ind}+\varepsilon_{p,i,t}$	(7)

${RD}_{p,i,t}=\alpha_{3}+\beta_{3}{LGD}_{p,t-1}+\lambda_{3}{EFD}_{p,i,t}+\chi_{3}{LGD}_{p,t-1}*{EFD}_{p,i,t}+\gamma_{3}{Control}_{p,i,t}+\delta_{t}+\theta_{p}+\tau_{ind}+\varepsilon_{p,i,t}$	(8)

where $Sub$ is the fiscal subsidy, measured by the logarithm of corporate fiscal subsidies. $Further, ERT$ is the corporate actual tax burden, following Hanlon and Heitzman [13]^[13]^, measured by
$\frac{income tax expense}{{pre\_tax accounting profit-deferred income tax expense}/{nominal tax rate}}$, $EFD$ is a dummy variable, representing the enterprise’s external financing dependence. Specifically, $EFD=\frac{CE-Adj\_CF}{CE},$ where $CE$ is measured by the cash paid by corporates to purchase and construct fixed, intangible, and other long-term assets: $Adj\_CF$ = operating cash flow + decrease in inventory + decrease in accounts receivable + increase in accounts payable. Finally, we use the median level of all enterprises in each industry to measure the external financing dependency of the industry that year. A corporate is defined as a high external financing dependence, which is higher than the median; otherwise, it is a low external financing dependence [14]^[14]^. The definitions of other variables are the same as in the baseline regression. If the coefficients of the interaction terms $\chi_{1}$, $\chi_{2}$, and$\chi_{3}$ are statistically significant, then LGD depends on Sub, ERT, and EFD, respectively, to have an impact on corporate R&D investment.

Table 5 reports the regression results of the mechanism test. The explained variables presented in columns (1), (2), and (3) in Table 5 are corporate R&D investment. The coefficients of LGD in these three columns are all significantly negative. This result indicates that, considering other conditions unchanged, LGD will reduce corporate R&D investment, which again verifies these conclusions. In column (1) of Table 5, the coefficient of the interaction term between LGD and fiscal subsidies is significantly positive, which implies that fiscal subsidies can alleviate the negative effect of LGD on corporate R&D investment. In column (2), the coefficient of the interaction term between LGD and the corporate actual tax burden is significantly negative. This fact indicates that LGD inhibits the corporate R&D investment by increasing the corporate actual tax burden. In column (3), the coefficient of the interaction term between LGD and external financing dependence is significantly negative. This result indicates that LGD inhibits enterprise’s R&D expenditures by reducing external financing of enterprises.

In summary, LGD affects corporate R&D investment by consuming regional fiscal resources, strengthening government tax collection efforts, and consuming regional credit resources.

| **Table 5. Mechanism analysis.** | | | |
| --- | --- | --- | --- |
| **Variables** | **Dependent variable: RD** | | |
|  | **(1)** | **(2)** | **(3)** |
| LGD | -0.007** | -0.001** | -0.002** |
|  | (-2.476) | (-2.100) | (-2.547) |
| Sub | 0.002*** |  |  |
|  | (8.080) |  |  |
| ERT |  | -0.005*** |  |
|  |  | (-5.274) |  |
| RZ |  |  | -0.001 |
|  |  |  | (-1.552) |
| LGD*Sub | 0.0003* |  |  |
|  | (1.951) |  |  |
| LGD*ERT |  | -0.002** |  |
|  |  | (-2.070) |  |
| LGD*RZ |  |  | -0.002* |
|  |  |  | (-1.907) |
| Other controls | Yes | Yes | Yes |
| Province fixed effects | Yes | Yes | Yes |
| Industry fixed effects | Yes | Yes | Yes |
| Year fixed effects | Yes | Yes | Yes |
| Observations | 11980 | 14133 | 14314 |
| Adj_R^2^ | 0.339 | 0.329 | 0.318 |
| **Notes:** ***, **, and * are indicate significant levels of 1%, 5%, and 10% levels, respectively. T-statistics are provided in parentheses below each coefficient estimate. Other controls include LnGDP, LnPop, SIZE, SOE, AGE, LEV, ROA, FIX, INV, and SHR1. | | | |

***4. Minor suggestions***

*Please supplement any unkind explanations and proofread grammatical errors.*

*For example, what are ST, *ST on page 9? What are i and t on page 10 (even though we can easily infer)? Sentences such as "The classic question in the study of the macroeconomic consequences of government debt is that how does the government debt affect economic growth." are wrong.*

**⇒Response:**

Thank you for pointing this out. **We have corrected it in the revised manuscript. For details, please refer to pages 9 and 10. The revised parts are also marked in red in the revised manuscript.**

1. “we excluded firms with abnormal financial or other conditions (ST firms), firms with risk of delisting stocks (ST^*^ firms), and suspended and delisted firms.”

2. “where $p$ is the province, $i$ is the firm, and $t$ is the time.”

3. “The normal question in the study of the macroeconomic consequences of government debt is how government debt affects economic growth.”

Thank you again for this opportunity to revise and resubmit our manuscript. We appreciate it and hope to hear from you soon.

Yours sincerely,

The Authors

1. Ansar A, Flyvbjerg B, Budzier A, et al. Does infrastructure investment lead to economic growth or economic fragility? Evidence from China. Oxford Review of Economic Policy. 2016; 32(3): 360-390.
2. Li H, Li L, Wu B, et al. The end of cheap Chinese labor. Journal of Economic Perspectives. 2012; 26(4): 57-74.
3. Fang L H, Lerner J, Wu C. Intellectual property rights protection, ownership, and innovation: Evidence from China. The Review of Financial Studies. 2017; 30(7): 2446-2477.
4. Fu T. The dilemma of government intervention in a firm's financing: Evidence from China. International Review of Financial Analysis. 2020; 71: 101525.
5. Allen F, Qian J Q J, Gu X. An overview of China's financial system. Annual Review of Financial Economics. 2017; 9: 191-231.
6. Organisation for Economic Co-operation and Development. OECD Reviews of Innovation Policy China. Organisation for Economic Co-operation and Development; 2008.
7. Hanley A, Liu W H, Vaona A. Credit depth, government intervention and innovation in China: Evidence from the provincial data. Eurasian Business Review. 2015; 5(1): 73-98.
8. Wooldridge, Jeffrey M. Introductory econometrics: A modern approach. Cengage learning; 2015.
9. Matsa, D. A. Capital structure as a strategic variable: Evidence from collective bargaining. The Journal of Finance. 2010; 65(3), 1197-1232.
10. Stock, James, and Motohiro Yogo. Asymptotic distributions of instrumental variables statistics with many instruments. Vol. 6. Chapter, 2005.
11. Larcker, D. F., Rusticus, T. O. On the use of instrumental variables in accounting research. Journal of Accounting and Economics. 2010; 49(3), 186-205.
12. Demirci I, Huang J, Sialm C. Government debt and corporate leverage: international evidence. Journal of financial economics. 2019; 133(2): 337-356.
13. Hanlon M, Heitzman S. A review of tax research. Journal of Accounting and Economics. 2010; 50(2-3): 127-178
14. Rajan RG, Zingales L. Power in a Theory of the Firm. The Quarterly Journal of Economics. 1998; 113(2): 387-432.
