# Supplementary material for: Crowding in or crowding out? How local government debt influences corporate innovation for China
Source: PLoS One. 2021 Nov 8;16(11):e0259452. doi: 10.1371/journal.pone.0259452 (PMC8575287; doi:10.1371/journal.pone.0259452)
Supplement: Attachment — Submitted filename: Referee Report.docx [file pone.0259452.s001.docx]

**0. Overall Statement**

This study examines the effects of local government debt on corporate R&D activities by focusing on the case of China. The authors find the crowding-out effects, and they argue that three channels (consumption of fiscal resources, strengthening tax collection efforts, consumption of credit resources) are channels that lead to these results. While the study tackles the interesting question with a valid empirical framework, I believe that there is much room for the improvement of the current document. Here, I mainly provide four suggestions.

**1. Institutional Background**

For international readers with various backgrounds, I recommend the authors elaborate on the institutional background of the study. For example, how and why does the corporate's innovative strategy require the local government's fiscal policies in China? For example, private enterprises’ R&D activities are not necessarily dependent on government funding in many developed countries.^[1]^

To do so, I suggest that the authors add a new section—Institutional Background—before (or after) the literature review to explain more about the detailed system in China. Providing anecdotes or examples can be beneficial. While some of the relevant information is included in the theoretical hypothesis in the current version, it would be effective to separate the hypothesis developments with institutional background.

**2. Causality argument**

Considering that the local government’s debts are non-random, inferring a causal linkage is difficult in this study. Of course, the authors conduct many robustness checks to support their results, which should be applauded for their efforts. Still, I have some reservations and request the following tests to improve the quality of estimation.

Dynamic panel analysis suggested by Arellano and Bond (1991) would be advantageous as the dataset is longitudinal and rich. This test is also helpful considering that R&D in the past may affect the current decision-making because R&D activities are performed on the basis of a long-term perspective.

While the authors apply the IV strategy to resolve the endogeneity,^[2]^ I am not convinced whether the current IV is satisfied with the exclusion assumption. There is no explanation about how the IV can resolve the endogeneity—such as reverse causality. For example, we can imagine that R&D activities may improve provincial economic conditions (such as employment, etc.) which may lead to affecting the debts of local governments, which the authors do not provide any explanation of how the IV can address this issue.

If the IV is not sufficiently convinced, I suggest that the authors should remove any causal argument in the current document. Instead, focusing on the correlation between the local governments’ debt and R&D activities can be better, which still contributes to the literature. (I admit that it requires substantial revisions, but it is worthwhile.)

**3. Mechanism analysis**

I believe that the mechanism analysis is not methodologically persuasive and only provides suggestive evidence. While the authors find correlations between the local government debts and fiscal variables by adopting these fiscal factors as a dependent variable, they do not give any concrete evidence that deteriorated fiscal conditions negatively affect R&D activities.

Here, two alternative approaches come to my mind. First, the authors may tone down their argument by removing any concrete conclusion. (In this way, the author can address the relationship between local government debts and R&D activities, and three channels can remain as possible discussions). Second, the author can conduct the regression by interacting the terms (between local government debts and fiscal variables) and R&D activities as a dependent variable. Although this is also not sufficient, it can provide a necessary condition for the authors’ argument.

**4. Minor suggestions**

Please supplement any unkind explanations and proofread grammatical errors.

For example, what are ST, *ST on page 9? What are i and t on page 10 (even though we can easily infer)? Sentences such as "The classic question in the study of the macroeconomic consequences of government debt is that how does the government debt affect economic growth." are wrong.

1. The authors explain “However, innovation, being a risky asset [10], often requires these policies from the government” in the part of Introduction. But readers who are not familiar with the Chinese context wonder why the corporate’s innovation requires such policies.
2. The authors argue: “From an exogeneity perspective, the maturity amount of the *t-1* Chengtou bond is determined by the time of bond issuance, so the exogeneity of the maturity amount of *the t-1* Chengtou bond is also satisfied.”
